# Financial Planning for Retirement: A Psychosocial Perspective

**DOI:** 10.3389/fpsyg.2017.02338

**Published:** 2018-01-24

**Authors:** Gabriela Topa, Gregg Lunceford, Richard E. Boyatzis

**Affiliations:** ^1^Faculty of Psychology, Universidad Nacional de Educación a Distancia, Madrid, Spain; ^2^Weatherhead School of Management, Case Western Reserve University, Cleveland, OH, United States

**Keywords:** retirement, financial planning, ideal self, personal vision, retirement planning

## Abstract

Retirement is a time of life that has grown ever longer in the developed world, and the number of pensioners has increased accordingly, questioning the strength of Social Security systems and the social safety net in general. Financial Planning for Retirement (FRP) consists of the series of activities involved in the accumulation of wealth to cover needs in the post-retirement stage of life. The negative short-, mid-, and long-term consequences of inadequate Financial Planning for Retirement do not only affect individuals, but also their extended families, homes, eventually producing an unwanted impact on the entire society. The Capacity-Willingness-Opportunity Model has been proposed to understand FPR, combined with Intentional Change Theory, a framework for understanding the process, antecedents and consequences of FPR. From this perspective, we propose this promising model, but there are a large number of variables that have not been included that offer novel ways to deepen our understanding of FPR. A focus on each dimension of the model, the role of age and psychosocial variables associated with demographic indicators such as gender, health status, and migration, allow us to provide a proposal of scientific advancement of FPR.

## Financial planning for retirement: a psychosocial perspective

From a societal standpoint, population aging in the developed countries has intensified pressure on public pension systems (Annink et al., 2016). It now seems clear that society will not be able to guarantee quality of life in retirement unless people save on their own behalf including private (i.e., corporate) pensions leading governments to adopt increasingly active policies designed to involve citizens in Financial Planning for Retirement (FPR). FPR consists of the series of activities involved in the accumulation of wealth to cover needs in the post-retirement stage of life. It is necessary because of the high, mid- and long-term, negative impact of poor planning (Choi and Jang, 2016; Ekici and Koydemir, 2016). At the same time, this activity is complex for several reasons. Firstly, most people do not possess the necessary knowledge to make optimal savings and investment decisions. Secondly, individual planning is subject to many factors, such as income, professional career, or health, which, moreover, interact with each other. Thirdly, people may experience anxiety and develop negative attitudes toward contemplating the latter stages of life and planning, ultimately avoiding FPR.

FPR was initially treated as a matter exclusively for economists, accountants and financial advisors. More recently, economists have found “a set of coherent explanatory constructs” useful to understand economic behavior (García-Gallego et al., 2017, p. 848) in psychological concepts. At the same time, in psychology, the importance of finances in retirement was admitted (Topa et al., 2011). Academics have progressively incorporated variables from other disciplines in their empirical studies, accumulating evidence for integrated models of retirement planning (Wong and Earl, 2009; Wang and Shultz, 2010). A wide range of personal resources has been explored as relevant predictor of successful adjustment to retirement (Leung and Earl, 2012). Despite this, empirical research on FPR has increased either without a theoretical model or with more general models, like the Theory of Reasoned Action.

In 2013, Hershey, Jacobs-Lawson, and Austin proposed a conceptual framework called the “Capacity-Willingness-Opportunity Model” to understand FPR. This model is promising for three reasons. It is specific, because it is designed to explain FPR. It is broad because it includes three dimensions with different types of variables. And it is procedural because it incorporates a temporal dimension, analyzing age and stage, and their interaction with the other facets of the model. As previous research suggested, different patterns of change should be considered when examining retirement outcomes (Wang, 2007).

Three dimensions—capacity, willingness and opportunity to plan for retirement—were proposed by Hershey and his colleagues in their model. Capacity refers to the cognitive factors and skills required to plan and save for retirement, distinguishing one person from the next. Among others, one's knowledge, skills, fluid, and crystallized intelligence, and psychological biases would likely influence the ability to plan and save (Resende and Zeidan, 2015). Meanwhile, willingness consists of the motivational variables that drive planning activities and saving. Hence, this dimension includes the motivational forces and the attitudinal and emotional factors that determine the likelihood that a given individual will begin planning and will sustain the activity over time. These factors are, among others, clarity and nature of one's financial goals, retirement-related fear and anxiety, perceived social norms, and self-image could be linked to the tendency to plan and save. Finally, the opportunity dimension acknowledges the existence of certain external influences, including environmental facilitators and constraints that affect effective financial tasking. Among others, the availability of voluntary retirement saving programs, tax incentives for saving, and financial advisors in the proximal environment would be associated with the tendency to plan and save.

Taken as a whole, the model is procedural. This means that the model holds a main assumption related to the continuity and strengthening of FPR during the course of adulthood. This turns our attention to the role of age in Hershey's model, which is somewhat complex. On the one hand, the continuity assumption implies that a stable pattern of entrenchment of capacity, willingness, and opportunities to plan and save could be expected.

It becomes a habit. Development of the value of FRP, like saving in general, is in part based on a person's ability to delay gratification for a long term goal or dream. On the other hand, this pattern of continuity is not immutable. At least three types of influences could lead to changes in FPR: normative age-related influences, normative history-related influences, and non-normative life events. Based on normative age-related influences, workers around 55 years old become more interested in financial planning than younger workers. In Europe, history-related influences could be exemplified by the pension system reform, which increased the population's awareness of the sustainability of future pensions. Finally, non-normative life events, such as major health problems, could interfere with FPR. Elimination of mandatory retirement age in various countries has changed a key benchmark of retirement. The development of a portfolio of part-time jobs and choosing which jobs need to be financially compensated opens new possibilities to reframe the concept of retirement. With lengthening of the years of quality and active living, people are searching for meaning and purpose beyond subsistence in the latter stages of life.

The empirical evidence supporting this model still is fragmentary and insufficient. Despite the fact that there is more than a decade's worth of empirical works that have examined partial aspects of the model (e.g., Hershey et al., 1998, 2007; Hershey and Mowen, 2000), there are no works that test the complete model. Emerging investigations in this regard have begun to appear (Jiménez et al., in press) but they can be clearly improved in design and data collection procedures (Topa et al., 2017b).

The evolution of one's life can be interpreted through the intentionality shown about adaptation, learning, and change. This is explained in Intentional Change Theory (ICT; Boyatzis, 2008). In this theory and the longitudinal research over the last 50 years about it and its components, explains that the first key discovery on the path to sustained, desired change is articulating a personal vision, or dream. This is not a set of goals, but something bigger and with a longer term framework. It is developed from the question, “If your life were perfect 10–15 years from now, what would your life and work be?” In contrast to goals or elements others have told you should be part of your future, your personal dream invokes neural networks, hormonal systems and psychological states that open the person to new ideas (Boyatzis et al., 2015). Other key elements in ICT are the resonant, trusting relationships that enable a person to explore and refresh in an iterative manner their dream and progress toward it. The role of such coaches or trusted advisors are another element key to a person developing a desired image of the latter stages of life and/or retirement, and an appropriate financial retirement plan.

Despite the fact that Hershey's model provides responses to a wide range of present questions about FPR, various untouched topics offer opportunities for moving forward in this domain. We will briefly discuss them, following the dimensions of the model.

## Further extensions

**Figure 1:**
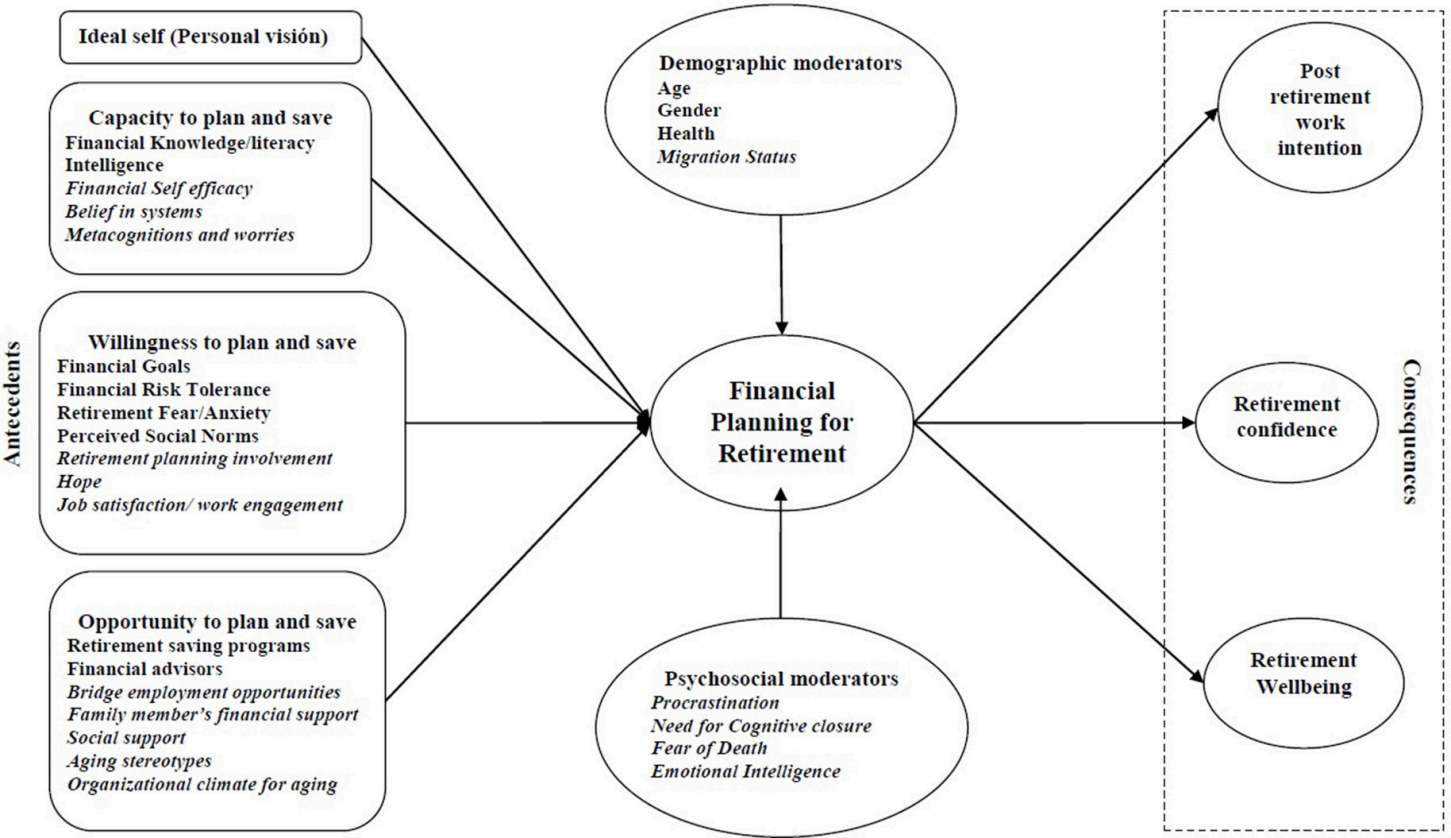
Expanded Model for FPR. Variables in italics reflect the extensions for the original Hershey's Model on FPR.

Related to capacity, additional variables should be considered (See Figure 1). First, the *Need for Cognitive Closure* refers to the individual's desire to obtain clear and definitive responses to a problem (Webster and Kruglanski, 1994). Empirical research reports significant differences between people with high and low need for cognitive closure regarding the quantity of information they process, the intensity of that information, the use of decision rules, and the level of self-confidence in their decisions. Accordingly, people with a high *Need for Cognitive Closure* are more likely to focus on information that is easy to process, reject complex or incomplete information, decide faster, have an urgent desire to achieve closure and retain it permanently. Its influence has been shown in a broad array of decision-making processes (Dolinski et al., 2016), but it has not yet been fully assessed in FPR. Due to the complexity and uncertainty entailed by FPR, as it implies the processing of complex information and the anticipation of needs with a high degree of uncertainty, we contend that *Need for Cognitive Closure* offers an interesting avenue to develop Hershey's model, as some empirical study has proposed (Topa et al., in press).

Secondly, FPR is a complex task that is often accompanied by worries. Thus, metacognition refers to beliefs about one's own cognitive system, the influences that affect it, attention and evaluation of the meaning of one's thoughts (Wells and Cartwright-Hatton, 2004). Due to the complexity of FPR and the worries associated with it, exploring the role of metacognitions is promising, as a recent empirical study showed (Kiso and Hershey, 2017). In particular, positive and negative beliefs about one's own worries, as well as cognitive trust and the self-attention, could play important roles in the relationships between capacity to plan and FPR. There is empirical evidence that supports the joint influence of metacognitions and worry in the development of anxiety in other contexts (Ryum et al., 2017). These same relations should be explored in the area of FPR, using validated instruments like the Meta-Cognitions Questionnaire 30 (MCQ-30; Wells and Cartwright-Hatton, 2004). The findings would help to understand why people consider it important to plan but do not carry out specific behaviors of saving and financial management.

As it relates to capacity, the development of one's retirement identity and financial self-efficacy is important in FPR. For past generations, FPR primarily focused on evaluating the adequacy of an individual's income and cash flow sources over a 5–10 year retirement lifespan. Improvements in lifestyle and medical technology have increased life expectancy and provided a 20–30 year increase in human longevity. In a related vein, Donaldson and colleagues showed that a higher personal sense of mastery, a variable near to self-efficacy, significantly mediated the relationships between pre-retirement planning and adjustment (Donaldson et al., 2010). FPR now requires careful evaluations and planning around an individual's future social emotional as well as economic needs.

Although workers may be old enough to fully retire in their 60's, they may not be ready to fully disengage from the workforce. For many, work provides value above and beyond economic gain. Such value includes a regular set of activities that provide time structure, status, identity, a sense of participation in a collective effort, and the opportunity to socialize with co-workers (Price et al., 1998). One's ability to form a view of their ideal self in retirement is a beneficial part of the retirement planning process (Lunceford, 2017).

Ideal self begins as a personal vision, or an image of what kind of person someone wishes to be (Boyatzis and Akrivou, 2006). In the context of retirement, ideal self can be what a person would like to accomplish after they choose to physically or psychologically reduce their commitment to their primary career. This may take the form of paid work or volunteer work on a part-time or full-time basis; it may include work in the same profession, development of a new skillset or vocation, or complete exit from the workforce to a life of leisure.

Ideal self is the driver of intentional change in one's behavior, emotions, perceptions, and attitudes (Boyatzis and Akrivou, 2006). It must also be an image of the kind of person they want to be in their later years. The Ideal Self involves a sense of hope about the future. Hope becomes an emotional tone for the future, instead of regret for a life not lived, fear of diminishing resources, or fear of death and the end game of life.

The development of the ideal self in the retirement planning process puts an image of the desired future at the center of the FRP, not merely financial analysis. It may be instrumental in addressing stress related to the alteration of career identity and finances in retirement. The formation of ideal self has a relationship to understanding an individual's intent to engage in bridge work (Lunceford, 2017). In addition, it positively influences well-being in the pre-retirement process (Lunceford, 2017). Measurement of the ideal self may be done with recent evaluation tools developed by Boyatzis et al. (2010).

The development of financial self-efficacy is also critical in the FPR process. Financial self-efficacy is a belief that one can be successful financially in certain situations. Self-efficacy is related to self-confidence, motivation, optimism, and the belief that one can cope with a variety of life's challenges (Bandura, 1997). People with high levels of self-efficacy believe they can perform well at specified tasks (Lown, 2011). In recent pre-retirement studies, financial self-efficacy was show to have a positive influence on career decision making, the formation of retirement confidence and well-being (Lunceford, 2017). They believe a desired future is somewhat in their control. In a related vein, the individual experience of aging could influence retirement planning in general and, specifically, FPR. As Heraty and McCarthy (2015) suggested, self-perceptions of aging could influence FPR among older workers, and also post-retirement employment options (Fasbender et al., 2014).

Related to willingness, various affective aspects and personality traits expand future research, based on complex relationships between emotions and decision-making (Hariharan et al., 2015). On the one hand, we should not forget that retirement marks the end of obligatory work and serves as a reminder of aging, failing health and, eventually, death. Unsurprisingly, then retirement can provoke unease, gloom and dread, and would be associated with fear of death, complex emotional phenomenon, which include apprehension both for oneself and for loved ones.

A growing body of empirical evidence shows that fear of death impinges on most spheres of life, and specifically on financial decisions. Despite this fact, only recent studies begin to consider the role of fear of death in explaining FPR (Topa et al., in press). Specifically, this study showed that fear of death acts as a moderator in the relationship between financial goals and retirement savings adequacy, mediated by financial behavior among clients of financial advisory firms. These results thus represent one step forward in the investigation of the influence of fear of death on people with strong anti-consumer attitudes, which are more likely to exercise tight control over their finances.

Related to the job attitudes-FPR relationships, previous studies indicate that retirement planning in general has negatively been predicted by job satisfaction and work involvement (Topa et al., 2009). As retirement is a crucial event in the life trajectory, work-related factors would be very relevant as antecedents of quitting one's job. On the one hand are positive attitudes toward the job, as strong engagement and high satisfaction would act as pull factors, influencing the individual decision to delay FPR (i.e., Zappalà et al., 2008). On the other, some studies note that work dissatisfaction influenced the decision to exit the organization but not the labor market, so there would no direct effect on FPR (Perera et al., 2015).

On the other hand, personal character traits also could influence FPR, even though little empirical research exists in this area (Rahimi et al., 2016). Procrastination consists of deliberately putting off or delaying an action which the subject nevertheless intends to take, especially where delay is likely to have adverse effects. This personality trait would affect FPR and outcomes, specifically due to the fact that many retirement-related goals are linked to a specific time horizon. This means that retirement goals seem to be imposed by the passage of the time and may have a specific time frame. Initial evidence in this direction has been recently provided by Topa and Herrador-Alcaide (2016), but only limited to a sample of workers between ages 45 and 63 employed by small- and medium-sized firms in Spain. Some present findings have been summarized in Table 1.

**Table 1:** Summary of recent studies with further extensions of the Hershey's Model.

**Study**	**Type of participants**	**Variables**	**Main findings**
Jiménez et al., in press	948 Spanish workers aged between 30 and 63. Cross sectional study	Three model dimensions-capacity, willingness and opportunities to plan and save + financial planning for retirement + moderator role of age in the relationships between antecedents and financial planning	Global support for the model. The younger participants showed a greater level of FPR if they were characterized by a high level of education. The interaction between both age and Psychological preparation for retirement and Retirement goals clarity failed to reach statistical significance
Palaci et al., 2017	280 participants aged between 45 and 63 years. Cross sectional study	Parental financial socialization, financial planning for retirement (FPR), financial literacy, financial planning decisions, and financial management	Parental financial socialization directly and indirectly influences FPR. Financial literacy, decisions about FPR and financial management mediated the relation between parental financial socialization and FPR
Topa et al., in press	Three-wave's study (*N* = 276) were 40-plus Spanish clients of financial advisory firms	The mediating role of financial behavior in the relationship between financial goals and retirement saving adequacy, and the moderating role of Fear of death	Relationship between financial goals and retirement saving adequacy is mediated by financial behavior. Fear of death moderates the financial behavior-retirement saving adequacy relationship
Topa et al., in press	Longitudinal study 272 adult non-students younger 40 years	The mediating role of investment advice use in the relation between investment literacy and financial management behavior among young adults. NCC moderates the relations between Investment advice use and financial management behavior	Employees with more investment advice use and characterized by high need for cognitive closure show a higher level of financial management behavior, both for the seizing and the freezing dimensions
Herrador-Alcaide et al., in press	Spanish aged workers (*N* = 452), who were in transition to retirement. Participants were still working at Time 1, and Time 2, while at Time 3 they will be retired during the past year	Combined influence of Financial Literacy, Financial Retirement Goals, Optimism to Retirement, Financial Risk Tolerance, and Commitment to financial planner (at Time 1), on Financial Management Practices (at Time 2); which will be associated with Financial Resources for retirement (at Time 3)	The main hypotheses have been fully supported by the Partial Least Squares analysis, predicting 36% of the Financial Management Practices' variance and 53% of the Financial Resources for retirement's variance
Topa et al., 2017b	A two wave longitudinal study with Spanish nurses older than 55 years (*N* = 132)	Retirement planning involvement, Retirement goals clarity, and Financial knowledge relationships with three types of retirement planning behaviors: Public Protection, Self-Insurance, and Self-protection	Statistically significant differences have been found: goal clarity in men positively affects all three planning dimensions, although it affects self-protection less. In women, goal clarity negatively influences public protection and strongly influences self-protection. For men, financial knowledge only influences public protection, in a negative sense, whereas for women, this relation does not exist and, in contrast, financial knowledge increases self-protection and self-insurance

Additionally, emotional intelligence has been widely researched over the past years due to its moderator role on the relationships between contextual antecedents and desirable personal outcomes. While the first conceptualization of emotional intelligence included appraisal and regulation of emotions, recent research has examined its incremental contribution, beyond personality features' contribution, to different indices of adaptation. As future planning and decision-making should be conceptualized as and adaptive process, also including a specific financial component, people who possess greater abilities in understanding and controlling their emotions could be also better in managing their FPR.

Concerning the opportunity dimension, a great amount of studies have relied on social support (Topa et al., 2017a), but the relationships with FPR of both organizational climate for older workers and aging stereotypes should also be taken into account. First, a healthy organization that provides older workers with motivational task design and generates an environment where successful aging is possible could positively influence not only employees' pre-retirement well-being (Guglielmi et al., 2016), but also their FPR. In a very basic sense, due to the fact that stress is resource consuming, healthier organizations seem to encourage their members to concentrate their efforts on future planning. Despite this promissory avenue, at present, no empirical research on these relationships was found.

Second, aging stereotypes widely spread both in organizational and in societal environments could undermine FPR. Despite the fact that direct relationships are difficult to conceive, links between negative stereotypes and FPR should be explored both through their negative effects on retirement self-efficacy (Valero and Topa, 2015)—which includes a financial dimension—or through their undermining future career prospects for older workers (Lytle et al., 2015). Moreover, while it would be expected that FPR would significantly increase among older workers, different exit pathways could be observed. Hence, the specific role of career transitions and bridge employment on FPR also deserves further exploration, as recent revision stated (Earl et al., 2015).

Few studies examine factors, especially non-financial, that lead to the intent to work in retirement (Kerr and Armstrong-Stassen, 2011) whether for pay or not, full time or part time or a portfolio of part time jobs. The recent study published by Cahill and colleagues with a large national-representative sample of older Americans, showed that bridge employment seems to be driven by other reasons than financial insecurity, both for those with little or medium financial assets. But, the same study found that those with less wealth seem to be more oriented to full time and wage and salary employment (Cahill et al., 2017). Additional studies could be beneficial to workers and employees. In many cases, societal norms place an unwelcomed expectation that individuals must disengage from work at a certain age (Topa et al., 2018). Given that individuals are living longer, age may be in conflict with the stage at which an individual is socially and emotionally ready to exit the workforce (Kim and Moen, 2001).

Anxiety about having to retire to conform to cultural expectations may limit employee morale and productivity. Also, the continued practice of expecting retirement based on age may promote negative stereotypes in the workplace that devalue meaningful contributors and place similar anxiety on their successors (Phillipson, 2012). Further study on factors that lead to post retirement work intention will inform employers on how to positively assist employees during late stage career transitions and retain key talent for longer periods. It will also provide advocacy and support for individuals who engage in the FPR process.

A recent study showed that helping workers understand and identify opportunities available to them in retirement has a positive relationship to their intent to work in retirement (Lunceford, 2017). Also, there is a relationship between an individual's level of financial self-efficacy and their intent to work in retirement (Lunceford, 2017). Given that a meaningful amount of education regarding retirement planning comes from employer sponsored plans the development of financial self-efficacy and a post retirement work plan concurrently could advance the model for FPR among the largest audience.

Moreover, related to the role of age in the model, a further development should consider that age is a multidimensional concept, which can be assessed under the chronological lens, but also as perceived, desired or group-referent age. However, at present, chronological age has been the only indicator used in FPR research (Hershey et al., 2013). In this sense, differentiating between functional, psychosocial, organizational, and lifespan age, as Scherbov and Sanderson (2016) suggested, allows us to explore their impact on FPR.

Further model development should explore remote antecedents, not yet included, which would help us to refine our understanding of FPR. Among others, parental financial socialization is one of the most widely assessed remote predictors of economical behavior among adolescents or college students (Trzcinska and Goszczynska, 2015), but there is a lack of studies applying it to older workers. Only one recent study (Palaci et al., 2017) considered its influence on FPR. Its findings show that parental financial socialization directly and indirectly influences FPR, through the mediation of financial literacy, financial planning decisions, and financial management. Moreover, parental financial behavior acts as a positive model for the development of financial literacy and skills and for decisions about FPR, opening future lines of early intervention.

Lastly, demographic characteristics, such as gender and health status, have been related to FPR (Zyphur et al., 2015). Despite which, due to their limited explanatory power *per se*, further research should be focused on gender-related variables, such as gender stereotypes, gender pay gap and gender differences in the Ultimatum Game behavior, in order to better understand why gender differences in FPR continue to emerge (Topa et al., 2017b).

In the same sense, the relationship between health and FPR may be complex. Firstly, we must remember that health can affect income, because illness limits access to better jobs and makes saving more difficult. Secondly, illness can influence subjective perceptions of income adequacy because poor physical and mental health can bias these affectively loaded measures. Thirdly, combinations of stressful events such as financial difficulties can make people more vulnerable when a negative event occurs. In this light, income may be more important for people who have suffered a decline in health than for those who have not (Han et al., 2015). Taking into account this complexity (Allen and Laborde, 2017), mutual influences among health status and FPR deserve further exploration.

Finally, a relevant demographic indicator, that has not been mentioned by Hershey et al. (2013), or even empirically explored, is migration status. Very little previous research has analyzed FPR among immigrants in Europe with large representative samples (Topa et al., 2012). Despite this, recent studies suggested that not only objective features, such as migration seniority, and also subjective social mobility, but also one's expectation about the possibility to move upward in the society (Huang et al., 2017), could influence immigrants' FPR. Both immigration status and acculturation have been showed as predictors of occupation health and long-term well-being for migrating workers (Vîrgă and Iliescu, 2017). Taking into account that migration is a global but enormously diverse phenomenon, specific patterns of FPR among temporal, circular and permanent immigrants need to be explored, mainly due to the fact that immigrants are frequently among the disadvantaged and less financially prepared groups.

## Implications

We hope that this brief review of unexplored topics illustrates both the complexity and the necessity of serious consideration of FPR. The benefits of more research will be societal, increasing publically funded costs of pensions and health care systems (Annoni and Weziak-Bialowolska, 2016). It could also include individual-level reasons, such as the strong influence of FPR on later financial and general well-being. The research should have sufficient sample size to allow for multivariate, causal and longitudinal analysis. Studies should be tiered, examining FRP of people in their 30's, 40's, 50's, 60's, and later.

Among other desirable consequences, more comprehensive models on FPR could encourage novel interventions for improving financial management (Harkin, 2017). It is likely that coaching people to their personal vision would help create a hopeful FRP, as well as voluntary participation in training programs, which in the long term guarantee updated skills for older workers (Sousa-Ribeiro et al., 2017). Such a plan might have more adherence and sustainable attention than plans developed through financial or another lens (Maurer and Chapman, 2017). Late career management should be considered as a novel field of expanded consequences of FPR (Wang and Wanberg, 2017).

There is also a social policy implication of expanded research. Considering that the worst blow “against human cognitive control abilities are retirement laws” (Hommel and Kibele, 2016, pp. 1184), we need to ask ourselves if the best FPR strategy could be to promote older people's work ability. Changing labor laws to remove retirement age restrictions might create a more accurate incentive to FRP. All of this would serve a purpose of helping people to work longer and guarantee long-term quality of life.

## Author contributions

All authors listed have made substantial, direct, and intellectual contribution to the present paper.

### Conflict of interest statement

The authors declare that the research was conducted in the absence of any commercial or financial relationships that could be construed as a potential conflict of interest.
